# Clinical Outcomes of Penetrating Keratoplasty and Descemet Stripping Automated Endothelial Keratoplasty in Asian Population with American Corneas

**DOI:** 10.3390/ijerph16224547

**Published:** 2019-11-17

**Authors:** Fang-Chi Hsiao, Po-Yuan Chen, Yaa-Jyuhn James Meir, Hsin-Yuan Tan, Ching-Hsi Hsiao, Hsin-Chiung Lin, David Hui-Kang Ma, Lung-Kun Yeh, Wei-Chi Wu, Hung-Chi Chen

**Affiliations:** 1 Department of Medicine, College of Medicine Chang Gung University, Taoyuan 33302, Taiwan; mtfredh@gmail.com (F.-C.H.); willy8421384213@yahoo.com.tw (P.-Y.C.); b0401018@cgmh.org.tw (H.-Y.T.); hsiao.chinghsi@gmail.com (C.-H.H.); hsinchiung@gmail.com (H.-C.L.); lkyeh@ms9.hinet.net (L.-K.Y.); weichi666@gmail.com (W.-C.W.); 2 Department of Ophthalmology, Chang Gung Memorial Hospital, Linkou 33305, Taiwan; hercats1@gmail.com (Y.-J.J.M.); davidhkma@yahoo.com (D.H.-K.M.); 3 Department of Biomedical Sciences, College of Medicine Chang Gung University, Taoyuan 33302, Taiwan; 4 Center for Tissue Engineering, Chang Gung Memorial Hospital, Linkou 33305, Taiwan; 5 Department of Chinese Medicine, College of Medicine Chang Gung University, Taoyuan 33302, Taiwan

**Keywords:** long term clinical outcomes, PK, DSAEK, imported corneas

## Abstract

To determine the comparative efficacy and safety of penetrating keratoplasty (PK) and Descemet stripping automated endothelial keratoplasty (DSAEK) in the Asian population receiving imported donor corneas, our single-center retrospective study provides analysis supporting the transition from PK to DSAEK in the Asian population using imported American donor corneas. We analyzed 259 patients with 241 and 57 cases of PK and DSAEK respectively during 2008 to 2017 using imported corneas at Chang Gung Memorial Hospital, Linkou, Taiwan. In terms of long-term graft survival analysis, there was no difference between PK and DSAEK (log-rank *p* = 0.386, HR = 0.920, 95% CI: [0.641–1.380]). However, Cox proportional regression analysis revealed that corneal survival rate of DSAEK group in the first 100 days after transplantation was inferior than that of PK group (log-rank *p* < 0.001, HR = 2.733, 95% CI: [1.501–4.977])]. Despite the inferior survival rate, there were significantly less neovascularization and Descemet membrane folds in the DSAEK group. Importantly, the non-complication rate of DSAEK was much higher than that of PK with significant difference (PK, 25.7% vs. DSAEK 42.0%, *p* = 0.022). Collectively, DSAEK is suggested as an alternative surgical modality in Asian patients using imported American donor corneas because of less complication, and no difference in long-term corneal graft survival rates between PK and DSAEK.

## 1. Introduction

Corneal transplantation is considered as the most common transplantation surgery in modern medicine [1]. Several surgical techniques have been proposed, including Descemet membrane endothelial keratoplasty (DMEK), Descemet stripping automated endothelial keratoplasty (DSAEK), and penetrating keratoplasty (PK) which are among the most common choices in corneal transplants [2,3].

Over the years, outcomes of the two techniques have been repeatedly compared in various aspects and the favorable results have been reported for DSAEK with domestic donors. DSAEK seems to result in refractive stability and reduced risk of graft rejection compared to PK [4,5]. Nonetheless, when it comes to foreign donors, there is a lack of studies with high level evidence supporting the beneficial result of transition from PK to DSAEK.

In Taiwan, the donation rate of cornea is not as high as that in Western countries [6]. During 1987–1999, there were 770 corneal transplantations with 93% imported corneal and 7% domestic corneal transplantation in a tertiary center of Taiwan. The indications were corneal scars (27.9%), regraft (21.0%), acute necrotizing and ulcerative keratitis (17.9%), pseudophakic or aphakic bullous keratopathy (17.6%), Fuchs’ dystrophy (4.5%), and keratoconus (2.5%) [7]. Though, the corneal donation rate gradually increased recently, importing corneas from foreign eye banks is still an important source of grafts [1,6].

There were some studies focusing on evaluating the changes in donor endothelial cell density (ECD) caused by precutting and long-distance transportation from overseas eye banks and concluded that the cell loss associated with precutting and the overseas transportation of corneal grafts on donor endothelial cell loss is “acceptable” [8,9]. However, there may be more factors contributing to the clinical outcome of imported corneal transplantation. For example, racial mismatch may influence the rejection rate of grafts. In multiple studies, including Price et al., Sugar et al., and Niziol et al. all found significant higher hazard ratio of rejection in African American recipients in corneal transplantation though the relatively smaller proportion of African American recipients in the PK studies [10,11,12]. Besides, surgeon learning curve may also influence the corneal grafts outcome when transitioning to a new type of keratoplasty. Pillar et al. had concluded that the rates of primary failure and disc dislocation for DSAEK decrease as surgeons gain experience with the procedure, and the number of functional grafts increases accordingly [13].

Corneal surgeons may still hesitate whether making the transition from PK to DSAEK because of graft dislocation, primary and late graft failures when receiving imported donor corneas in Asian countries [7,13]. In this review, we analyzed the associations between the risk factors and clinical outcomes of keratoplasty using imported donor corneas in a Taiwanese tertiary medical center. Our data analyzing the long-term corneal graft survival as well as complication rate of PK and DSAEK from Asian patients receiving imported donor corneas.

## 2. Material and Methods

### 2.1. Subjects

Our study followed the principles of the Declaration Helsinki and was approved by the institutional review board (IRB) and ethics committee of Chang Gung Memorial Hospital, Linkou, Taiwan with IRB number of 201800675B0. The IRB waived the need for informed consents because of the retrospective nature of this study and our promise to keep the identifying information removed and delinked. Participants’ information was collected from clinical records registered for the imported corneas at Chang Gung Hospital during January 2008 to January 2017. Corneas were imported from the Cincinnati Eye Bank, Indiana Lions Eye Bank, and New Jersey Eversight. Data were collected from medical records including age, sex, medical history, preoperative evaluation, operation notes, and outpatient department follow-up survey.

The survival period of the corneal graft was defined as the time between the date of surgery to the first documented complication in medical records such as corneal edema, corneal haze, and corneal opacity etc. Complications which were not relieved by treatment in consecutive two months were regarded as corneal rejection. All patients undergoing PK or DSAEK were included. Patients who did not complete at least two weeks of follow-up were excluded. Corneal grafts with survival time less than 14 days which were treated as acute graft rejection (primary failure) were excluded in further complication analysis as well.

### 2.2. Interventions

All donor corneas were provided by American Eye Banks in adherence to EBAA medical standards approval of the Eye Banking Committee of the American Academy of Ophthalmology. The containers were maintained at 2–8 °C in validated shipping containers throughout the entire shipment process. Upon arrival at Chang Gung Hospital, tissues were checked and documented by our technicians, including the date and time of arrival, condition of the shipping container, and status of each viewing chamber. Further, the corneas were matched with individual recipients. Patients and procedures’ information was collected from electronic clinical records during Jan 2008 to Dec 2017. PK were performed by eight and DSAEK were by seven corneal surgeons in Chang Gung Hospital, and using standard surgical technique of PK and DSAEK. All patients underwent a complete ophthalmic examination before keratoplasty, and medical history was documented. Defined postoperative examination were performed at 1, 3, 6, 9, 12, 18 and 24 months and annually thereafter.

### 2.3. Statistical Analysis

Statistics analysis was using IBM SPSS Statistics 23.0.0.0 software (IBM Corp., Armonk, NY, USA). Descriptive statistical analysis of the frequency of PK and DSAEK each year, recipients and donors’ categorized data, recipients and donors’ continuous data, indications as well as complications were reported as frequencies. Median and interquartile range was applied to present the properties and dispersion of the recipients and donors’ continuous data. 

Kaplan-Meier survival analysis with log-rank test was used to compare the corneal survival of patients undergoing PK or DSAEK. To conduct statistical analysis on the subgroup result, univariate Cox regression analyses were first conducted for each continuous or categorical variable to evaluate the significance, for which *p*-value is <0.15 in univariate analysis were to further evaluate the significance with corrected factors in multivariate Cox regression analyses. Cox proportional regression analysis was used to obtain the specific hazard ratio of corneal survival because our study aimed to investigate association rather than estimation of the probability of corneal rejection [14,15,16]. Chi square test was used in complications analysis. Statistical significance was defined as *p*-value < 0.05.

The definition of categorized diseases was as following. Hypertension: blood pressure > 140/90 mmHg since the patients’ hypertension was all diagnosed before 2016. Diabetes mellitus: fasting plasma glucose ≥ 126 mg/dL at least two times or HbA1c > 6.5%. Hyperlipidemia includes hypercholesterolemia: fasting serum total cholesterol 200 mg/dL, and/or LDL cholesterol ≥ 130 mg/dL, and hypertriglyceridemia: serum triglyceride > 150 mg/dL. Heart related diseases includes arrhythmias, ischemic heart disease, heart failure, chronic heart disease, pericardial and valvular disease. Cataract was diagnosed by ophthalmologist with eye exam. Glaucoma: progressive optic neuropathy with visual filed defect includes open angle and angle closure glaucoma. All categorized diseases were extract from ICD codes. Anterior chamber clarity was based on slit lamp examination before corneal transplantation. 

## 3. Result

### 3.1. Demographics

Among the 323 cases of transplantation using imported cornea between January 2008 and January 2017 at Chang Gung Hospital, there were 241 cases of PK including 14 cases of acute graft rejection and 9 lost-follow cases after transplantation, 57 cases of DSAEK including 6 cases of primary failure and 1 lost-follow case after transplantation, 12 cases of other lamellar keratoplasty, and 13 cases without complete recipient clinical data, which were thus excluded. 

The frequency of PK and DSAEK for every year from 2007 to 2017 is presented in Table 1. The mean follow-up of 218 cases of PK was 43.5 ± 26.0 months (range: 2.0–113.8) while that was 34.7 ± 26.9 months (range: 1.0–92.6) in 50 DSAEK cases. The corneal donors were 90% Caucasians, while other 10% was recorded as “other” in ethnicity category from the eye bank documents. The detail data of recipients and donors who underwent PK or DSAEK are shown in Table 2.

In the aspect of the indications of PK and DSAEK (Table 3), the most common indication of PK was graft rejection 42.1% (107/241), followed by aphakic bullous keratopathy, and pseudophakic bullous keratopathy (ABK/PBK) 16.2% (39/241). Trauma-derived category containing corneal scar caused by trauma, by physical, and chemical harm occupied 14.9% (36/241). Bacteria, fungus, and Acanthamoeba infection were categorized as non-viral infection which comprised 4.6% (11/241). In the PK cases of viral keratopathy, there were 4.1% (10/241) in which pathogens were all herpes simplex virus. As for the survival analysis of different indications, there is no significant difference in regraft and ABK/PBK of PK group (96 vs. 34 patients, log-rank *p* = 0.35) nor the difference in BK/PBK and other indications of DSAEK group (22 vs. 28 patients, log-rank *p* = 0.83). The survival curve of BK/PBK in PK and DSAEK group also revealed no difference (34 vs. 22 patients, log-rank *p* = 0.19). 

### 3.2. Graft Survival of PK and DSAEK

**Figure 1:**
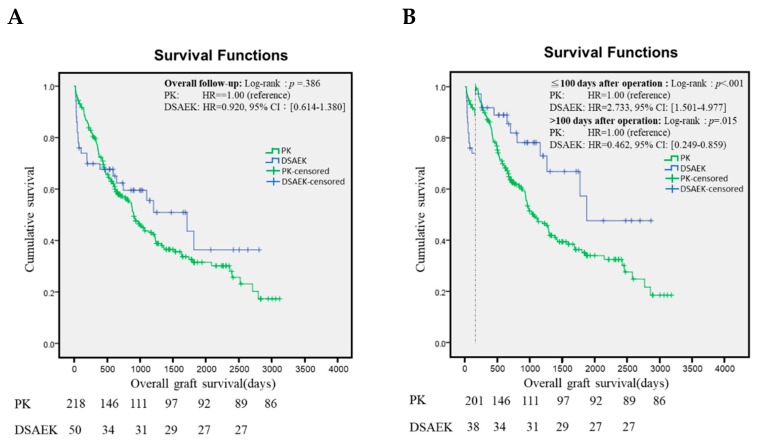
PK and DSAEK graft survival. (**A)** Long-term survival analysis of PK (green) and DSAEK (blue). (**B**) Cox proportional regression analysis stratified by 100 days follow-up. PK = penetrating keratoplasty. DSAEK = Descemet stripping automated endothelial keratoplasty. HR = hazard ration. CI= confidence interval.

Three-year survival rate of PK using imported cornea was 43.5%, while three-year survival rate of DSAEK was 59.5%. In the long term graft survival analysis, there was no difference between PK and DSAEK (log-rank *p* = 0.386, HR = 0.920, 95% CI: [0.641–1.380]), as shown in Figure 1A. Cox proportional regression analysis revealed that corneal survival rate of DSAEK group in the first 100 days after transplantation was inferior than that of PK group (log-rank *p* < 0.001, HR = 2.733, 95% CI: [1.501–4.977]), while the corneal survival of DSAEK group after 100 days of transplantation was superior than that of the PK group (log-rank *p* = 0.015, HR = 0.462, 95% CI: [0.249–0.859]), as shown in Figure 1B. 

### 3.3. Recipients Risk Factors of PK

Various recipient conditions were analyzed for the corneal survival after PK (Table 4). In univariate cox analysis, we found that recipients without glaucoma history had better outcome (*p* = 0.008). The recipients with discernable anterior chamber before surgery also had superior corneal survival (*p* = 0.039). For the risk factors *p*-value < 0.15 were further analyzed in multivariate Cox regression analysis. Though diabetes mellitus, hyperlipidemia, glaucoma, and regraft showed higher hazard ratio, they were not statistically significant by multivariate Cox regression analysis. Pre-transplantation blood survey of WBC, segment and lymphocyte were all unrelated to corneal graft survival.

### 3.4. Complications

Post-transplantation complications are shown in Table 5 over mean 43.5 months following up in PK groups and 34.7 months in DSAEK group. The most common complication was cornea edema (38.5% and 50.0% in PK and DSAEK respectively). We observed DSAEK group showed lower neovascularization after transplantation (PK, 14.2% vs. DSAEK, 2.0%, *p* = 0.016). There was difference in occurrence of Descemet membrane folds in two keratoplasty (PK, 26.6% vs. DSAEK, 8.0%, *p* = 0.005). Also, we found that keratic precipitates occurrence seems to be lower in DSAEK (PK, 11.9% vs. DSAEK, 4.0%, *p* = 0.098). As for infection, there was no difference in non-viral infection. HSV infection rate was higher in DSAEK (PK, 0% vs. DSAEK, 2.0%, *p* = 0.036). In our data, CMV infection presented higher in DSAEK (PK, 0.4% vs. DSAEK, 5.3%, *p* = 0.004). In three cases of CMV infection in DSAEK, one suffered from complications of recurrence, another endothelitis, and the other iritis. Other complications such as glaucoma, subconjunctival hemorrhage, superficial punctate keratitis, pigment epithelial detachment, corneal ulcer, microcystic corneal edema, peripheral anterior synechiae, hypotony, and stitch-related abscess revealed no statistically difference between PK and DSAEK. Importantly, the non-complication rate of DSAEK was far higher than PK showing significant difference (PK, 25.7% vs. DSAEK 42.0%, *p* = 0.022). As for the graft dislocation which is limited to DSAEK, there was a 28.0% graft dislocation rate after DSAEK, of which 13 cases (26.0%) had air rejection and 1 case (2.0%) had graft reattached later.

## 4. Discussion

As mentioned above, DSAEK presented a lesser complication rate compared to PK in overall following time (mean follow-up time, PK: 43.5 ± 26.0, DSAEK: 34.7 ± 26.9 months). Nonetheless, the survival rate of DSAEK was inferior to that of PK in the first 100 days after transplantation. The clinical outcome of our study seems to have some discrepancy compared to other studies, which used domestic corneal grafts as their study material.

To be specific, multiple studies reported better corneal survival time and clinical outcomes of DSAEK (or EK) [17,18,19,20,21,22,23,24], for example, Tan D.T. et al. and Fuest M. et al. [17,25]. Besides, Price M.O. et al. also stated that the median 3-year survival rate for DSAEK and PK was 46% and 51% respectively (*p* = 0.33) in Fuch’s [23]. Similarly, in the retrospective study by Anshu et al. 60 cases DSEK under failed PK, the graft survival rates were reported as 98%, 90%, 81%, and 74% at 1, 2, 3, and 4 years, respectively [26]. The result slightly exceeded the previously reported survival rates for PK graft. However, not all studies showed overwhelming benefit of DSAEK. Wang F et al. reported no significant difference in graft survival rate of EK versus PK after failed PK in a meta-analysis of various reports [19]. 

In our study, the benefit of DSAEK was not as prominent as that of the aforementioned reports. We made a detailed analysis to find which factors would contribute to this. The primary indication of PK revealed no significant difference in regraft and ABK/PBK group (96 vs. 34 patients, log-rank *p* = 0.35) as well as regraft plus PBK and other indications (130 vs. 90 patients, log-rank *p* = 0.16). The primary indication of DSAEK also revealed no significant difference in ABK/PBK and other indications (22 vs. 28 patients, log-rank *p* = 0.83). As for the ABK/PBK in PK and DSAEK group, no significant difference was found (34 vs. 22 patients, log-rank *p* = 0.19), but the survival curve crossed at 500–600 days showing similar trend as Figure 1A. It seems that the indication of the PK or DSAEK was not the primary factor that affect the graft survival.

The patients’ information, underlying disease and pre-operation laboratory testing in the PK group also showed no remarkable effect on graft survival. Probably, racial disparities might arbitrarily affect the outcome of PK and DSAEK, while we did not have enough evidence to conclude that. Therefore, two factors could attribute to this result, i.e., surgeon experience and longer preservation-to-operation time. 

First, for all new surgical techniques, surgeons would only achieve optimal level of competence after certain amount of time and experience [27]. Hence, surgeon learning curve may play a major role in the expected outcome of DSAEK. Chang Gung medical center has dedicated in corneal transplantation for more than 40 years, and PK dominated the treatment of choice even until recent years. In contrast, DSAEK was not introduced to our medical center until June 2009 and the case number up to now is still less than that of PK. Chen et al. have indicated surgeon learning curve as an important factor influencing the outcome of corneal transplant [28]. Similarly, Miriam et al. also further conducted a 8-year national prospective cohort study which included 2139 recipients of 2615 endothelial grafts (DSAEK and DSEK), registered by 85 surgeons in Australia, examining longitudinal graft survival with Kaplan–Meier survival analyses and Cox proportional hazards regression [29]. Conclusively, the survival of the first 56 registered grafts was significantly poorer than that of the subsequent grafts, when data were combined for all surgeons. Besides, the frequency of primary non-functioning graft was also significantly less likely to be reported for grafts performed by surgeons with more than 56 registered cases experience. In fact, when compared with surgeons with less workload (less than 56 cases in the 8 years of study), those with higher workload achieved significantly better graft survival. The learning curve would only be less apparent after 57 or more cases during their study period. In our study the overall DSAEK case number was still under the learning curve (57 cases), and therefore it is possible that the benefit of DSAEK including less complication and longer graft survival period may be more prominent with more surgeon experience and workload.

Besides, longer preservation-to-operation time may also have an adverse effect on graft of DSAEK. Although Lekhanont et al. concluded that the outcomes of DSAEK performed with internationally shipped donor cornea (mean shipping time: 9.52 days) were acceptable and the percentages of endothelial cell density (ECD) were comparable to those domestic grafts [30], the postoperative mean ECD at 6 month, 1, 2, 3 and 5 years of that study was still lower than the count of domestically shipped corneas. This implied that during the prolonged shipment though the percentages of endothelial cell loss after procedure were comparable to those achieved in Western series, the absolute count of endothelial cells was still impaired during the shipping period. In the studies of Sanjay V. Patel, he proposed that endothelial keratoplasty offered better outcome compared with PK in visual outcomes and a smaller incision [31]. However, the DSAEK manipulations of the fragile donor tissue caused significant donor endothelial cell trauma. As a result, donor endothelial cell loss was much higher during the first month after endothelial keratoplasty, and the cell loss speed would only rapidly decrease beyond 6 months after the procedure compared to PK [31]. In our studies the mean preservation day of our graft was 9.78 days, which was slightly longer than all of the studies mentioned above. With less initial absolute endothelial cell count and more decline in endothelial cells after DSAEK procedures compared to PK, it is plausible to assume that the initial downslope of endothelial cell is more evident in DSAEK that used imported donor corneas, veiling part of DSAEK potential survival benefit.

Interestingly, our data which showed that the survival curves of PK and DSAEK came cross each other at approximately 500 days after surgery may be compatible with Dickman et al. findings, in their research in 2016. It demonstrated similar phenomenon that the survival curves of PK and endothelial keratoplasty for PBK cross each sur approximately 42 months in Kaplan-Meier survival plot [32]. We speculate that there might be some specific adverse factors causing graft failure in the first 3 months post-DSAEK. The mechanism behind it may be due to the rapid decrease of endothelial count immediately after the DSAEK procedure. Many studies including Patel, Price et al., and Van Rooij et al. have observed this phenomenon [31,33,34,35].

In univariate Cox analysis of PK, we found that anterior chamber clarity in pre-operation evaluation showed positive impact on corneal survival after PK. Anterior chamber clarity before operation suggests less infection possibility and lower inflammation around the cornea which contribute to less immune reaction. Anterior chamber associated immune deviation is inhibited by inflammation cytokine such as IL-1 and TNF-α [36]. Thus, anterior chamber inflammation causes higher corneal graft failure and rejection rate [37]. Though higher peripheral blood lymphocyte percentage may suggest higher possibility of systemic inflammation, the specific correlation of peripheral blood lymphocyte percentage and anterior chamber lymphocyte number needs more evidence to clarify.

In our study, HSV and CMV infection rate in DSAEK was higher than that in PK. This may be attributed to small number of DSAEK group and CMV infection patients in both PK and DSAEK group in our study (PK:1 case, DSAEK:3 cases). One of the three cases in DSAEK group was recurrence of CMV endotheliitis. Besides, in recent years, an increasing number of cases of CMV-associated anterior segment inflammation in otherwise healthy individuals have recently been reported [38,39,40]. This apparent increase may be due to the improved availability of molecular techniques using PCR. More evidence is needed to determine whether patients who underwent DSAEK are more susceptible to CMV infection than PK.

## 5. Conclusions

Overall, DSAEK presented less complication over mean 34.7 ± 26.9 months follow-up than PK over mean 43.5 ± 26.0 months of following up. However, corneal survival rate of DSAEK group in the first 100 days after transplantation was inferior to that of the PK group. Ophthalmologists should thoroughly discuss with the patients the choice of transplantation technique, especially when using imported foreign corneal graft. We recognize the limitations of this study are similar to those of most retrospective and registry studies, including no comparison of endothelial cell number after shipping, loss of endothelial cells during follow-up and best corrected visual acuity before and after surgery. In conclusion, for an average of three-year post-keratoplasty follow-up, the results of this study suggest a significantly less complication rate in Asian population undergoing DSAEK than PK using imported American donor corneas.

## Figures and Tables

**Table 1:** The frequency of penetrating keratoplasty (PK) and Descemet stripping automated endothelial keratoplasty (DSAEK) for every year from 2007 to 2017.

	2008	2009	2010	2011	2012	2013	2014	2015	2016	2017-Jan
PK	15	33	22	37	34	30	30	22	16	2
DASEK	0	3	9	5	5	9	7	13	6	0

**Table 2:** Summary of recipients and donors’ information.

	PK				DSAEK			
	*N*	%	Median	Interquartile Range	*N*	%	Median	Interquartile Range
**Recipient**								
Ethnicity:Taiwanese	238	98.8%			57	100.0%		
Age (years)	241		61.0	[53.0–70.5]	57		67.0	[60.0–73.0]
Gender:male	135	56.0%			25	43.9%		
Operation site:OD	129	53.5%			25	43.9%		
Hypertension	73	30.3%			22	38.6%		
Diabetes mellitus	35	14.5%			8	14.0%		
Hyperlipidemia	13	5.4%			4	7.0%		
Heart related disease ^1^	28	11.6%			6	10.5%		
Cataract	162	67.2%			20	35.1%		
Glaucoma	46	19.1%			14	24.6%		
Anterior chamber clarity	114	47.3%			-	-		
Recipient cornea cut (mm)	219		8	[8.00–8.25]				
Graft cornea size (mm)	207		7.5	[7.50–8.00]				
White blood cell (1000/ul)	236		6.7	[5.7–8.0]	56		6.2	[5.4–7.15]
Lymphocyte (%)	196		30.8	[25.1–37.1	42		31.9	[26.1–38.9]
Segment cell (%)	196		60.2	[52.9–66.2]	42		59.2	[51.3–66.6]
Graft survival time (day) *	229		617	[279–1232]	50	-	602	[73–1163]
**Donor**								
Ethnicity:Caucasians	124	89.9%			39	95.1%		
Age (years)	138		65.0	[59.0–69.0]	41		61.0	[50.5–67.5]
Gender:male	70	50.7%			22	53.7%		
Death to preservation (h:min)	96		07:36	[5:29–10:51]	35		07:18	[5:16–9:47]
Death to operation (day)	137		9	[8,9,10]	40		9.5	[8.0–11.0]
Endothelial cell density (/mm^2^)	135		2525	[2347–2688]	40		2642	[2446–2853]

^1^ including arrhythmias, ischemic heart disease, heart failure, chronic heart disease, pericardial and valvular disease. * data collected from the last follow up when conducting this study.

**Table 3:** Indications of PK and DSAEK.

	PK		DSAEK	
Indication	Frequency	Percent%	Frequency	Percent%
Graft rejection	107	44.4%	11	19.3%
Aphakic bullous keratopathy/PBK	39	16.2%	26	45.6%
Trauma-derived	36	14.9%	1	1.8%
Non-viral infection	11	4.6%	0	0%
Viral keratopathy	10	4.1%	2	3.5%
Cornea degeneration	6	2.5%	4	7.0%
Endothelial dystrophy	1	0.4%	6	10.5%
Other	31	12.9%	7	12.3%
Corneal opacity	17	7.1%	1	1.8%
Corneal edema	10	4.1%	6	10.5%
Corneal ulcer	2	0.8%	0	0%
Exposure keratitis	2	0.8%	0	0%
Total	241	100%	57	100%

PBK = pseudophakic bullous keratopathy.

**Table 4:** Univariant and multivariant Cox regression analysis of risk factors of PK recipients.

Risk Factors (Categorical Variant)	Univariate Analysis		Multivariate Analysis		
	*p*-Value	HR	*p*-Value	HR	95% CI for HR
Sex					
Male		1.000			
Female	0.768	0.951			
Operation site					
OD		1.000			
OS	0.199	1.114			
Hypertension					
No		1.000			
Yes	0.800	0.955			
Diabetes mellitus					
No		1.000			
Yes	0.115	1.422	0.344	1.519	[0.639–3.609]
Hyperlipidemia					
No		1.000			
Yes	0.058	1.801	0.566	1.340	[0.493–3.639]
Heart related diseases					
No		1.000			
Yes	0.871	1.021			
Cataract					
No		1.000			
Yes	0.572	0.905			
Glaucoma					
No		1.000			
Yes	0.008	1.688	0.507	1.323	[0.578–3.028]
Anterior chamber clarity					
No		1.000			
Yes	0.039	0.702	0.894	0.961	[0.538–1.720]
Regraft					
No		1.000			
Yes	0.106	1.325	0.391	1.314	[0.704–2.453]
Donor Ethnicity					
Caucasian		1.000			
Non-Caucasian	0.625	0.831			
**Risk Factors (continual variant)**					
Recipient					
Age	0.873	1.001			
Recipient cornea cut (mm)	0.561	1.217			
Graft cornea size (mm)	0.352	1.276			
White blood cell (1000/µL)	0.634	1.021			
Segment cell (%)	0.114	0.985	* 0.118	0.974	[0.943–1.007]
Lymphocyte (%)	0.063	1.021	0.072	1.033	[0.997–1.070]
Donor					
Age	0.295	0.988			
Death to preservation time	0.740	1.000			
Death to operation time	0.280	1.103			
Graft endothelial cell density	0.014	1.001	0.214	1.001	[1.000–1.002]

HR = hazard ratio. CI = confidence interval. * Blood segment percentage has strong relation to lymphocyte percentage, so these two variants were respectively put in the multivariant cox regression model with the other 4 factors.

**Table 5:** Complications of PK and DSAEK.

Complications	PK		DSAEK		
	*N*	%	*N*	%	*p*-Value
Edema	84	38.5%	25	50.0%	0.137
Glaucoma	2	0.9%	2	4.0%	0.105
Neovascularization	31	14.2%	1	2.0%	* 0.016
Subconjunctival hemorrhage	2	0.9%	0	0.0%	0.497
Descemet membrane folds	58	26.6%	4	8.0%	* 0.005
Superficial punctate keratitis	30	13.8%	4	8.0%	0.270
Keratic precipitates	26	11.9%	2	4.0%	0.098
Pigment epithelial detachment	4	1.8%	0	0.0%	0.335
Ulcer	13	6.0%	0	0.0%	0.088
Microcystic corneal edema	17	7.8%	7	14.0%	0.166
Peripheral anterior synechiae	12	5.5%	0	0.0%	0.090
Bullae	15	6.9%	6	12.0%	0.224
Hypotony	3	1.4%	0	0.0%	0.404
Hypopyon	5	2.3%	0	0.0%	0.280
Stitch-related abscess	3	1.4%	0	0.0%	0.404
Non-viral infection	1	0.5%	1	2.0%	0.253
CMV infection	1	0.5%	3	6.0%	* 0.004
HSV infection	0	0.0%	1	2.0%	* 0.036
Graft dislocation	-	-	14	28.0%	-
Re-bubbling	-	-	13	26.0%	-
No complication	56	25.7%	21	42.0%	* 0.022

PK = penetrating keratoplasty. DSAEK = Descemet stripping automated endothelial keratoplasty. * indicates *p*-value <0.05.
